# Supplementary material for: Structural Analysis and Substrate Specificity of D-Carbamoylase from Pseudomonas
Source: BioTech (Basel). 2024 Oct 3;13(4):40. doi: 10.3390/biotech13040040 (PMC11503299; doi:10.3390/biotech13040040)
Supplement: Supplementary file 1 [file biotech-13-00040-s001.zip › biotech-13-00040-s001.pdf]

BioTech

MDPI

Supplementary Material

Structural analysis and substrate specificity of D-carbamoylase from the bacterium *Pseudomonas*

Marina Paronyan, Haykanush Koloyan, Hovsep Aganyants, Artur Hambardzumyan, Tigran Soghomonyan, Sona Avetisyan, Sergey Kocharov, Henry Panosyan, Vehary Sakanyan and Anichka Hovsepyan

Melting points were determined on a Boetius PHMK 76/0904 hot-stage microscope (Germany). The IR spectra were recorded on a Nicolet Thermo FT-IR spectrometer (USA) in thin films (Nujol).^{1}H and ^{13}C NMR spectra were recorded on a Varian Mercury-300 VX spectrometer (USA) in DMSO-d_6/CCl_4, 1:3. Chemical shifts are reported in δ values (ppm) relative to tetramethylsilane as internal standard. Coupling constants (*J* values) are given in Hertz (Hz). The signals are reported as follows: s (singlet), d (doublet), dd (double doublet), spt (septet), m (multiplet), br. (broad). All chemicals used were of analytical or reagent grade. Carbamoyl derivatives were prepared using the D-amino acids from Reanal (Hungary).

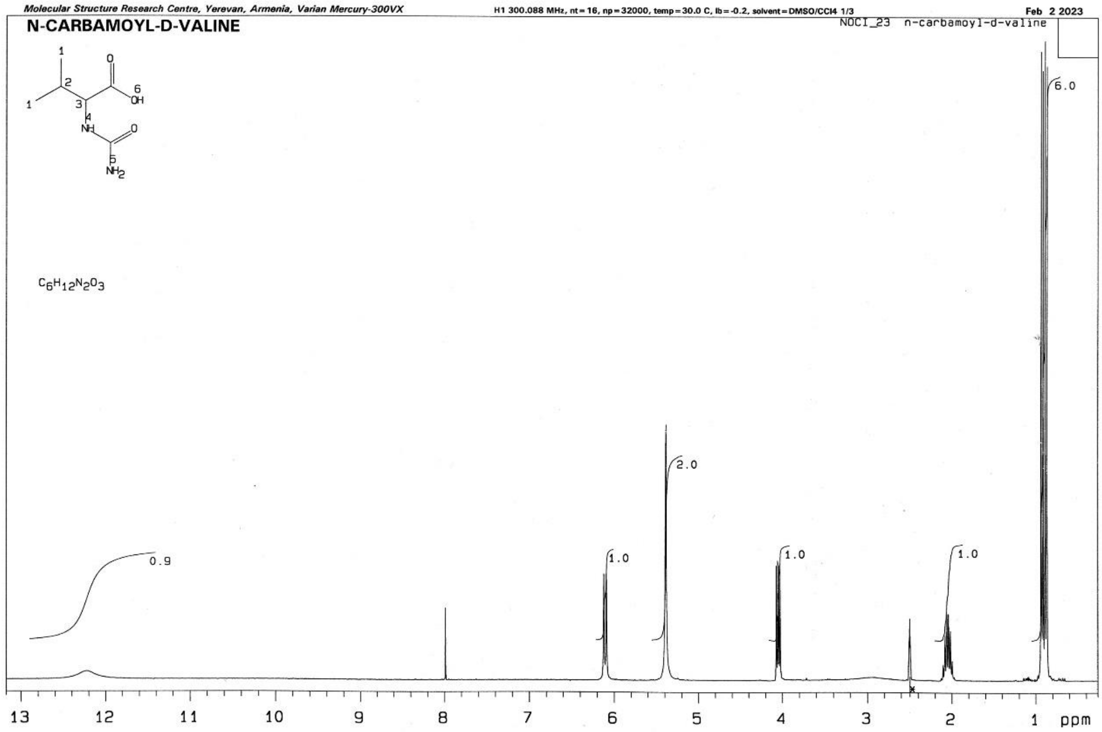

A

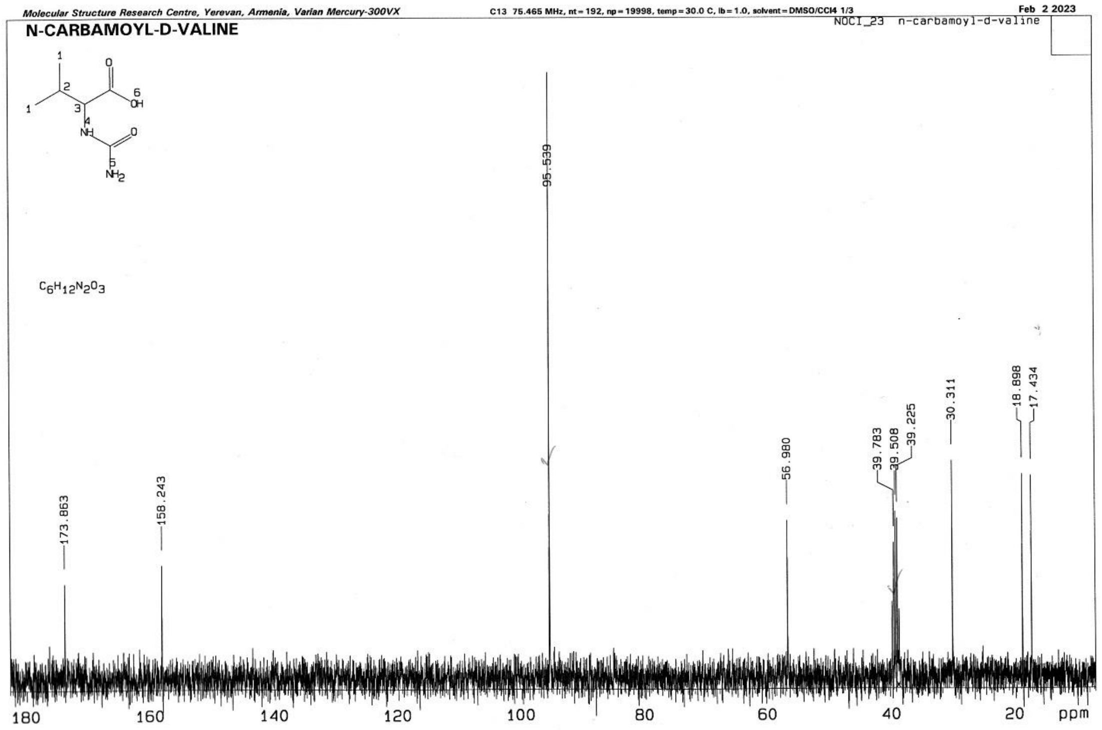

B

Figure S1. ^{13}C (A) and ^{1}H (B) NMR spectra of N-carbamoyl-D-valine.

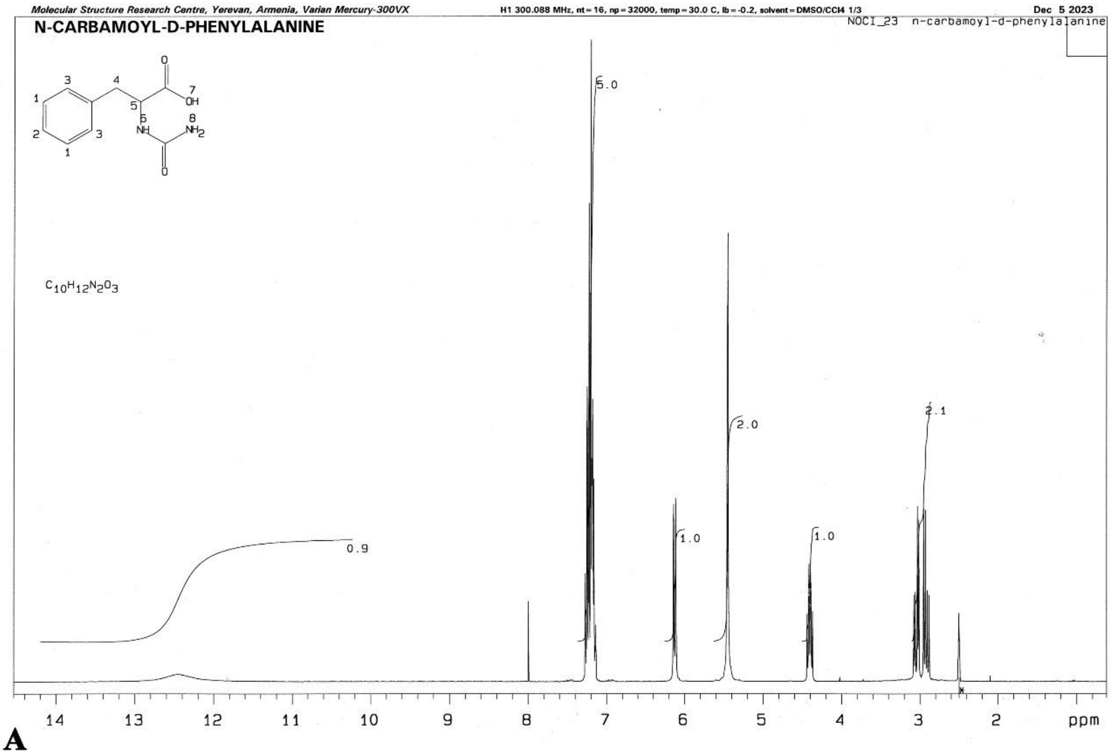

A

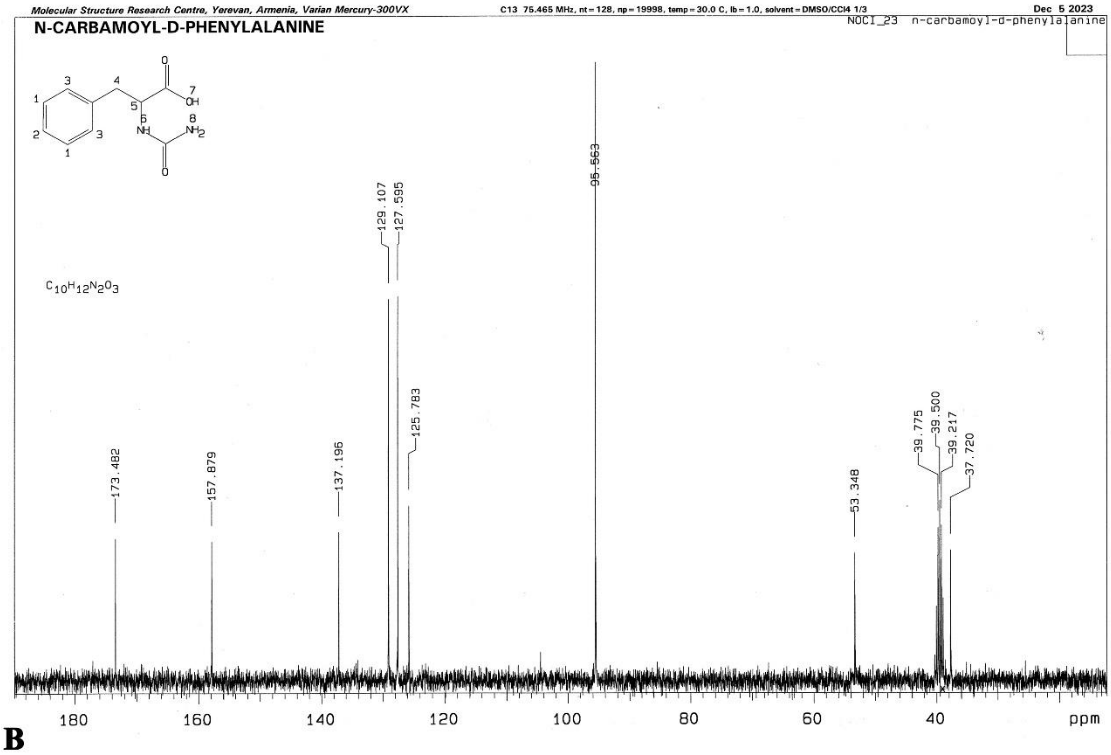

B

Figure S2. ^{13}C (A) and ^{1}H (B) NMR spectra of N-carbamoyl-D-phenylalanine.

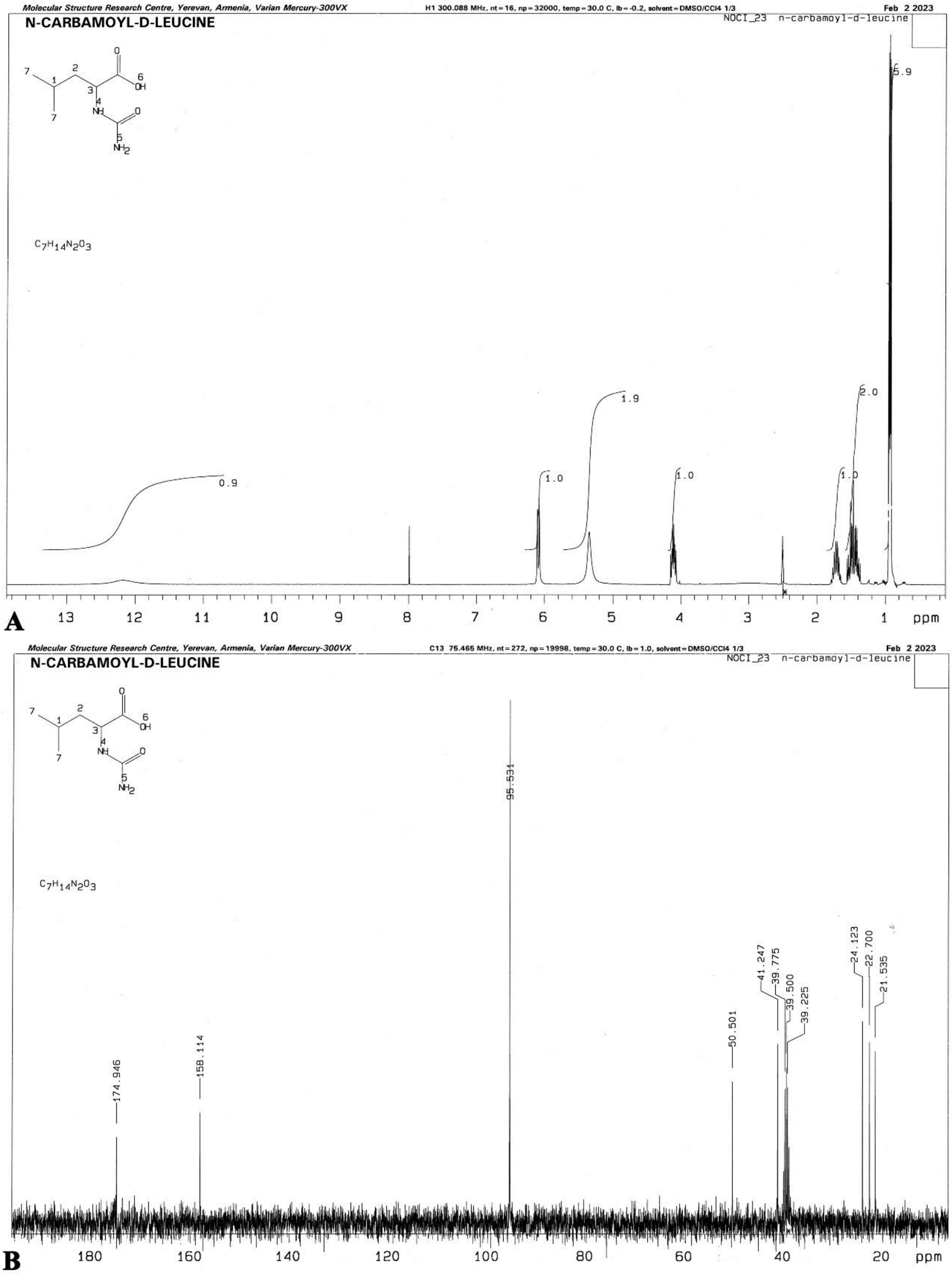

Figure S3. ^{13}C (A) and ^{1}H (B) NMR spectra of N-carbamoyl-D-leucine.

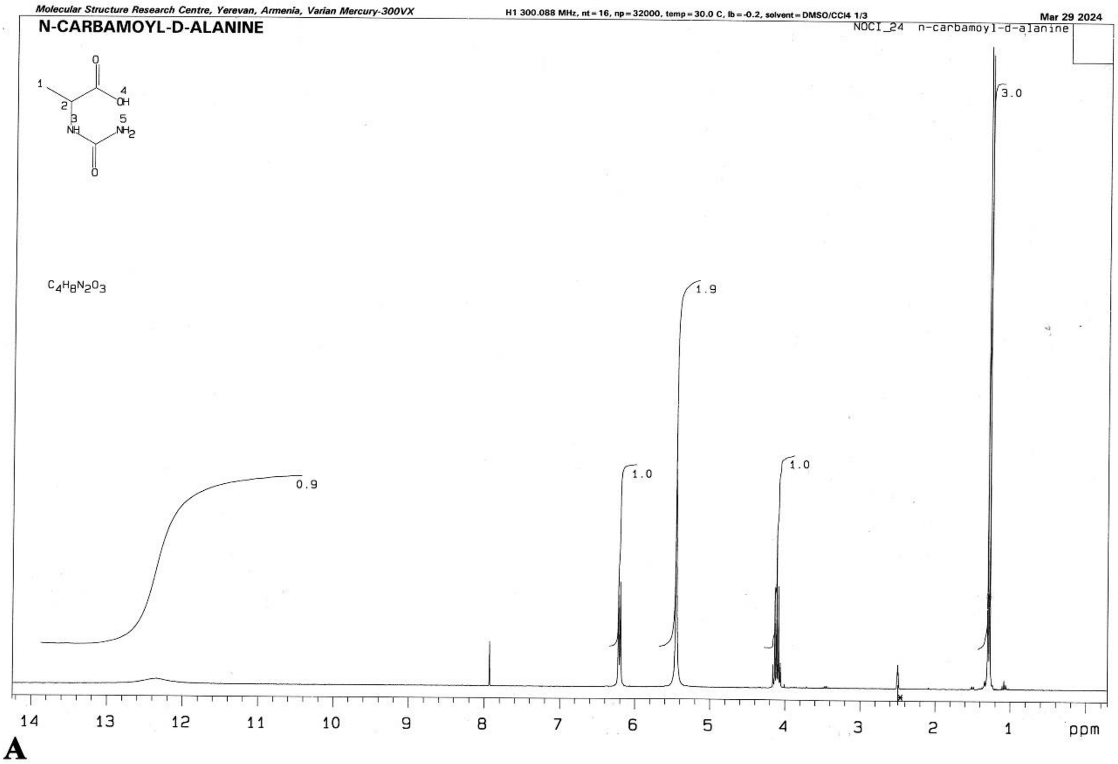

A

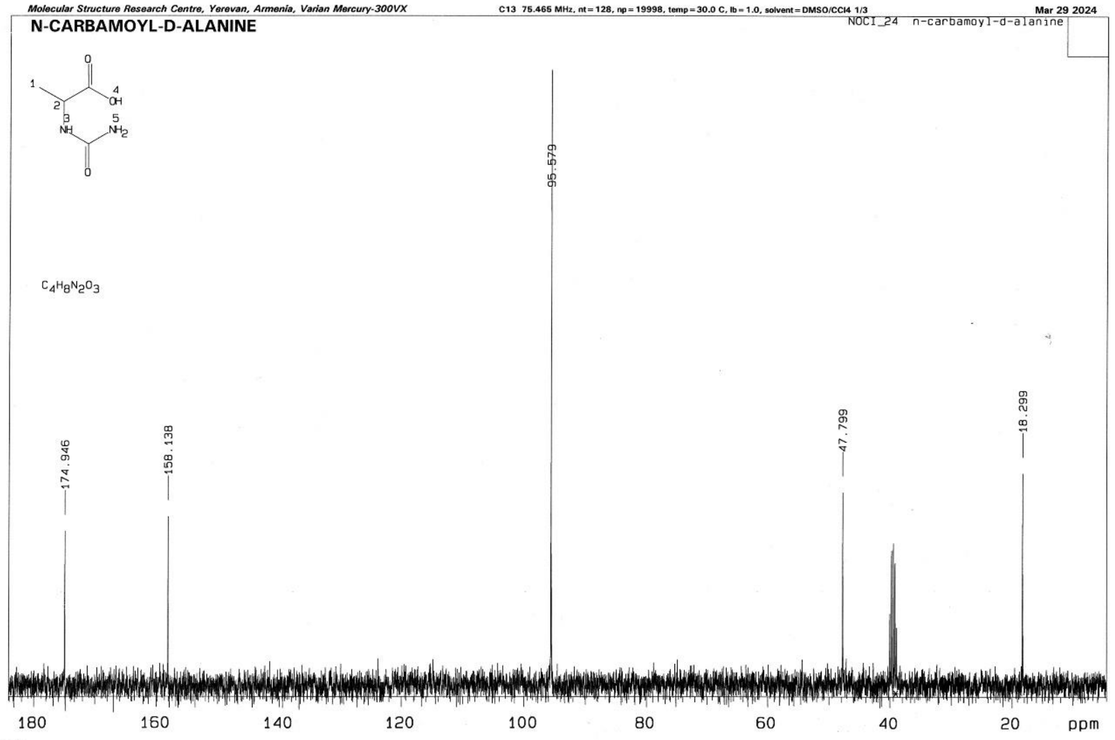

B

Figure S4. ^{13}C (A) and ^{1}H (B) NMR spectra of N-carbamoyl-D-alanine.

General procedure for synthesis of carbamoylamino acids.

The mixture of equimole amounts of amino acid and sodium cyanate (NaOCN) in water was kept at a room temperature during 75-80 hours (**1, 2, 4**) or at 100 °C for 4 hours (**3**). Then pH of reaction mixture was adjusted to 2 – 3 with conc. HCl. The separated solid was filtered and recrystallized. From filtrate additional amount of product was obtained after concentrating at reduced pressure.

Synthesis of D-3-Methyl-2-ureidobutanoic acid.

From 1.5 g (12.8 mmol) D-Val and 0.832 g (12.8 mmol) NaOCN 1.42 g of **1** was obtained (yield 69.27%). M. p. 225 – 226 °C (EtOH-H_2O, 5:1). IRS ν_{max}, cm^{-1}: 3453, 3291 (NH_2, NH), 1686 (C=O), 1633 (NH def., amide I), 1557 (C=O amid.), 1387, 1380 (doublet, isopropyl group). ^{1}H NMR, δ, ppm: 0.88 (d, 3H, *J* 6.8 Hz, CH_3), 0.93 (d, 3H, *J* 6.8 Hz, CH_3), 2.05 (spt.d, 1H, *J* 6.8, 4.9 Hz, CH_3C**H**CH_3), 4.0 (dd, 1H, *J* 9.1, 4.9 Hz, C**H**NH), 5.39 (br., 2H, NH_2), 6.11 (d, 1H, *J* 9.1 Hz, NH), 12.22 (br., 1H, COOH). ^{13}C NMR, δ, ppm: 17.4 (CH_3), 18.9 (CH_3), 30.3 (CH_3**C**HCH_3), 57.0 (NCH), 158.2 (NCO), 173.9 (O=C-O). $C_6H_{12}N_2O_3$. M 160.18. Analysis: calcd./found (%) N 17.49/17.27.

Synthesis of D-2-Carbamoylamino-3-phenylpropanoic acid.

From 1.25 g (7.57 mmol) D-Phe and 0.492 g (7.57 mmol) NaOCN 1.18 g of **3** was obtained (yield 74.68%). M.p. 212 °C (H_2O). IRS ν_{max}, cm^{-1}: 3452, 3350 sh., 3297, 3265sh. (NH_2, NH), 1695 (C=O), 1636 (NH def., amide I), 1566 (C=O amid., arom.). ^{1}H NMR, δ, ppm: 2.93 (dd, 1H, *J* 13.7, 7.0 Hz, CH_2), 3.03 (dd, 1H, *J* 13.7, 5.4 Hz, CH_2), 4.41 (ddd, 1H, *J* 8.1, 7.0, 5.4 Hz, CH), 5.45 (br. 2H, NH_2), 6.12 (d, 1H, *J* 8.1 Hz, NH), 7.13 – 7.27 (m, 5H, C_6H_5), 12.47 (br., 1H, COOH). ^{13}C NMR, δ, ppm: 37.7 (CH_2), 53.3 (CH), 125.8 (CH, *p*-C_6H_5), 127.6 (2CH, *o*-C_6H_5), 129.1 (2CH, *m*-C_6H_5), 137.2 (C*ipso*, C_6H_5), 157.9 (NCO), 173.5 (O=C-O). $C_{10}H_{12}N_2O_3$. M 208.22. Analysis: calcd./found (%) N 13.46/13.38.

Synthesis of D-2-Carbamoylamino-4-methylpentanoic acid.

From 0.5 g (3.81 mmol) D-Leu and 0.248 g (3.81 mmol) NaOCN 0.62 g of **2** was obtained (yield 93.37%). M.p. 233 – 234 °C (H_2O). IRS ν_{max}, cm^{-1}: 3458, 3350, 3303 (NH2, NH), 1686 (C=O), 1634 (NH def., amide I), 1573 (C=O amid.), 1407, 1377 (isopropyl group). ^{1}H NMR, δ, ppm: 0.93 (d, 3H, *J* 6.6 Hz, CH_3), 0.94 (d, 3H, *J* 6.6 Hz, CH_3), 1.37 – 1.55 (m, 2H, CH_2), 1.63 – 1.81 (m, 1H, CH_3C**H**CH_3), 4.11 (ddd, 1H, *J* 9.1, 8.6, 5.2 Hz, C**H**NH), 5.35 (br., 2H, NH_2), 6.09 (d, 1H, *J* 8.6 Hz, NH), 12.15 (br., 1H, COOH). ^{13}C NMR, δ, ppm: 21.5 (CH_3), 22.7 (CH_3), 24.1 (CH), 41.2 (CH_2), 50.5 (CHNH), 158.1 (NCO), 174.9 (O=C-O). $C_7H_{14}N_2O_3$. M 174.20. Analysis: calcd./found (%) N 16.08/15.92.

Synthesis of D-2-Ureidopropanoic acid.

From 1.00 g (11.22 mmol) D-Ala and 0.73 g (11.22 mmol) NaOCN 1.28 g of **4** was obtained (yield 86.40%). M.p. 201.5 – 202 °C (EtOH-H_2O, 2:1). IRS ν_{max}, cm^{-1}: 3455, 3309. 3350 sh., 3250 (NH_2, NH), 1700 sh., 1692 (C=O), 1633 (NH def., amide I). ^{1}H NMR, δ, ppm: 1.29 (d, 3H, *J* 7.2 Hz, CH_3), 4.11 (dq, 1H, *J* 7.7, 7.2 Hz, CH), 5.45 (br., 2H, NH_2), 6.20 (d, 1H, *J* 7.7 Hz, NH), 12.36 (v.br., 1H, COOH). ^{13}C NMR, δ, ppm: 18.3 (CH_3), 47.8 (CH), 158.1 (NCO), 174.9 (O=C-O). $C_4H_8N_2O_3$.M 132.12. Analysis: calcd./found (%) N 21.21/20.97.

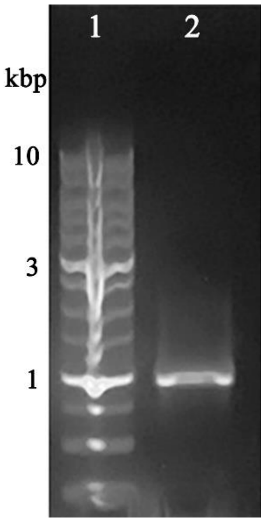

Figure S5. 0.8% agarose gel electrophoresis of the colony PCR product.

Lines 1 − DNA ladder, 2 − D-carbamoylase gene of *Pseudomonas* sp. strain KNK003A with size 936 bp.

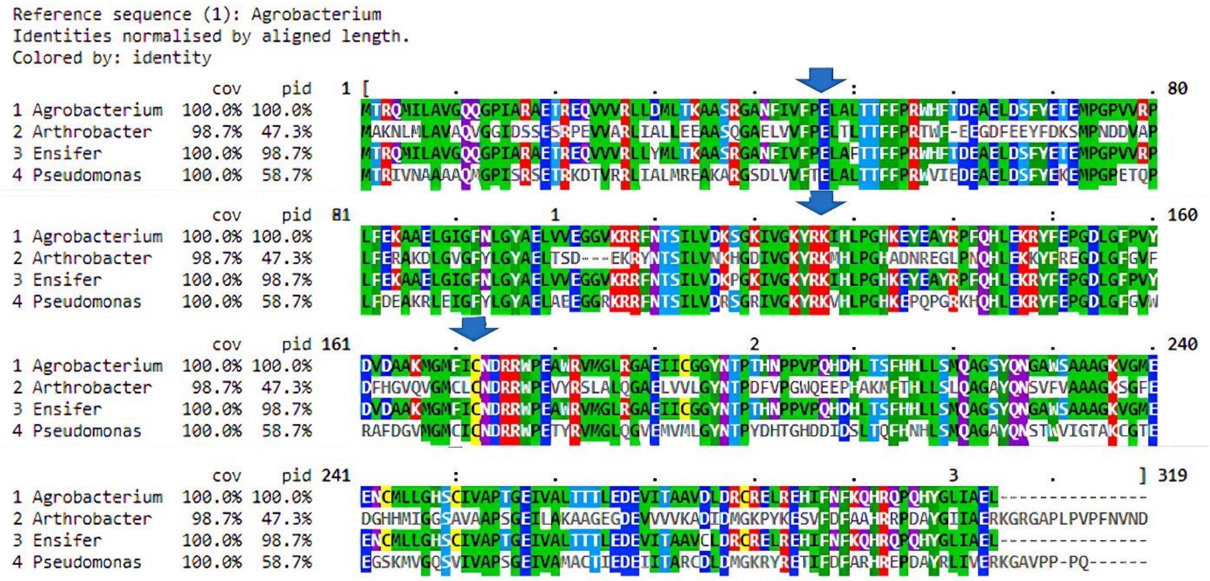

Figure S6. Multiple sequence alignment of D-carbamoylases of *Ensifer adhaerens* S-5, *Pseudomonas* sp. strain KNK003A, *Agrobacterium* sp. strain KNK712 and *Arthrobacter crystallopoietes* DSM 20117 strains. Blue arrows indicate conserved catalytic triad.

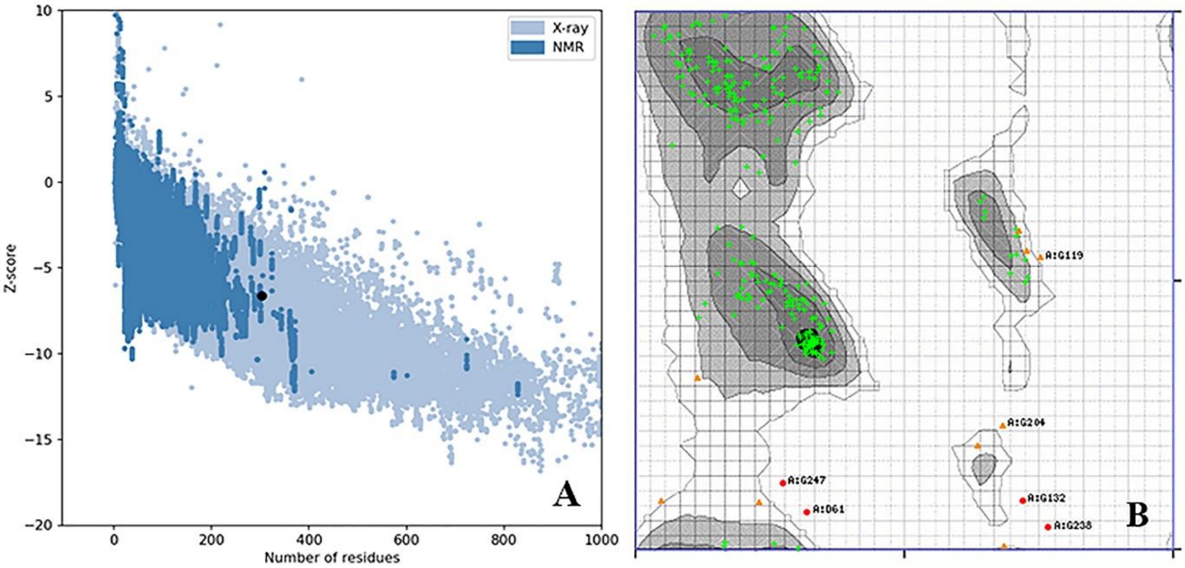

Figure S7. ProSa Web z-score plot for *Pseudomonas* sp. strain KNK003A D-carbamoylase model (**A**) and Ramachandran plot for modeled *Pseudomonas* sp. strain KNK003A D-carbamoylase (**B**). Regions colored Black, Dark Grey, Grey and Light Grey represent highly preferred conformations.
